# Development and validation of prediction models for the discharge destination of elderly patients with aspiration pneumonia

**DOI:** 10.1371/journal.pone.0282272

**Published:** 2023-02-24

**Authors:** Yoshito Hirota, Jung-ho Shin, Noriko Sasaki, Susumu Kunisawa, Kiyohide Fushimi, Yuichi Imanaka

**Affiliations:** 1 Department of Healthcare Economics and Quality Management, Graduate School of Medicine, Kyoto University, Kyoto, Japan; 2 Department of Health Policy and Informatics, Graduate School of Medical and Dental Sciences, Tokyo Medical and Dental University, Tokyo, Japan; Kyushu University, JAPAN

## Abstract

**Background:**

Discharge planning enhances the safe and timely transfer of inpatients between facilities. Predicting the discharge destination of inpatients with aspiration pneumonia is important for discharge planning. We aimed to develop and validate prediction models for the discharge destination of elderly patients with aspiration pneumonia.

**Methods:**

Using a nationwide inpatient database, we identified aspiration pneumonia cases for patients aged ≥65 years who had been admitted to hospital from their home or from a nursing home between April 2020 and March 2021. We divided the cases into derivation and validation cohorts according to the location of the admitting hospital. We developed two prediction models by dividing the cases based on the patient’s place of residence prior to admission, one model to predict the home discharge of cases admitted from home and the other to predict the home or to a nursing home discharge of cases admitted from a nursing home. The models were internally validated with bootstrapping and internal-externally validated using a validation cohort. Nomograms that could be used easily in clinical practice were also created.

**Results:**

The derivation cohort included 19,746 cases admitted from home and 14,359 cases admitted from a nursing home. Of the former, 10,760 (54.5%) cases were discharged home; from the latter, 7,071 (49.2%) were discharged to either home or a nursing home. The validation cohort included 6,262 cases admitted from home and 6,352 cases admitted from a nursing home. In the internal-external validation, the C-statistics of the final model for the cases admitted from home and the cases admitted from a nursing home were 0.71 and 0.67, respectively.

**Conclusions:**

We developed and validated new prediction models for the discharge of elderly patients with aspiration pneumonia either to home or to a nursing home. Our models and nomograms could facilitate the early implementation of discharge planning.

## Introduction

The number of deaths from aspiration pneumonia in Japan nearly doubled from 2010 to 2020, with 22,066 deaths in 2010 to 42,746 deaths in 2020 [1], and is expected to triple in the decade to 2030 as Japanese society ages [2]. Aspiration pneumonia is a pulmonary inflammation caused by the inhalation of oropharyngeal secretions containing colonized pathogenic bacteria [3]. A diagnosis is made when a patient with known or strongly suspected dysphagia and aspiration shows radiographic evidence of pneumonia [4]. Aspiration pneumonia has unique epidemiological features and outcomes that differ from community-acquired pneumonia [5]. Compared to those with community-acquired pneumonia, patients with aspiration pneumonia tend to be older and have lower functional status, more comorbidities, longer stays in hospital, and higher mortality [5].

While the discharge destinations for patients with aspiration pneumonia vary from country to country, patients in Japan are mainly discharged to their home, a nursing home, or a hospital with long-term care beds. Approximately half of all elderly patients with aspiration pneumonia are discharged to nursing homes [6], while one-third are discharged to a place other than where they lived before admission [7]. Discharging patients to the latter requires further planning and coordination [8,9]. Discharge planning enhances the safe and timely transfer of inpatients between different places, thus reducing hospital lengths of stay, resource use, and readmissions [10–12]. Accurately predicting the discharge destinations of inpatients with aspiration pneumonia is thus a vital issue in discharge planning. Although home discharge prediction models have been developed for surgical patients and stroke patients [13,14], similar attempts have not been reported for patients with aspiration pneumonia.

Several prior studies have identified key factors related to the home discharge of elderly patients with pneumonia, including aspiration pneumonia [6,15,16]. These studies showed that impaired physical function at discharge, low albumin level at admission, artificial nutrition, and respiratory care such as oxygen administration and sputum suctioning at discharge were negatively associated with home discharge. The generalizability of these results, however, is limited since most [6,15] were conducted using a single center and a small sample size. Moreover, some of the factors used in these studies were measured at discharge [6,16], and thus the results could not be used to predict home discharge based solely on patient characteristics at admission. Therefore, the objective of the current study was to develop and validate prediction models for the discharge destination of elderly patients with aspiration pneumonia using baseline patient characteristics that could be determined at the earliest stages of admission.

## Materials and methods

### Data source

In conducting the study, we used Diagnosis Procedure Combination (DPC) data from the database of the DPC research group, which is funded by the Ministry of Health, Labour and Welfare. The database contains DPC data collected across Japan from approximately 1,200 voluntarily participating hospitals [17]. The DPC data are used not only for reimbursement but also for Ministry of Health, Labour and Welfare surveys to improve medical systems and policies. Described in detail elsewhere [18], the DPC data contain administrative claims data and discharge clinical summaries that include the following: hospital identifier, age, sex, body mass index (BMI), history of drug prescriptions, medical procedures performed, length of stay, and discharge destination, as well as major diagnoses, the cause of admission, the most and second-most medical-resource-intensive diagnoses, comorbidities at admission, and complications after admission, which are recorded using the International Classification of Disease, Tenth Revision (ICD-10) codes.

### Study population

The study examined the cases of patients aged ≥65 years who were admitted to and discharged from the study hospitals between April 1, 2020 and March 31, 2021; who were recorded as having aspiration pneumonia (ICD-10 code, J69.x) in the major diagnosis and as the cause of admission; and who had lived either at home or in a nursing home immediately prior to admission. We excluded cases with missing data on the predictors. In conducting the study, we followed the recommendation of Steyerberg et al. that the data be split according to time or location for internal-external validation in order to show the external validity of the model [19]. Thus, the cases in our study were divided into derivation and validation cohorts in a ratio of approximately 3: 1 according to whether the hospital was located in east or west Japan. Consequently, the derivation cohort included the cases admitted to and discharged from study hospitals located in the eastern half of Japan from the Kinki region, while the validation cohort included cases admitted to and discharged from study hospitals located in the western half of Japan from the Tyugoku region. We further separated the cases into two groups in order to develop our two prediction models, depending on the patient’s residence immediately prior to admission: one group consisted of cases admitted from home; the other consisted of cases admitted from a nursing home.

### Outcomes

The outcome for cases admitted from home was home discharge, while the outcome for those admitted from a nursing home was discharge to their home or to a nursing home (hereinafter referred to as “home/nursing home discharge”). In Japan, nursing homes can be divided into two main types. The first type is comprised of facilities that provide medical care, such as integrated facilities for medical and long-term care, and healthcare facilities for the elderly requiring long-term care. The second type consists of nursing homes that do not provide medical care, such as special nursing homes, as well as communal living for the elderly with dementia. Since patients who have been cured of aspiration pneumonia often live for long periods of time in nursing homes that provide no medical care, such homes were defined as “nursing homes” in this study.

### Predictors

With respect to the predictors, no variable selection was performed since the deletion of variables, even non-significant ones, could impair predictive accuracy [20]. Following a review of the existing studies, we established the final prediction models with 19 predictors associated with outcomes that had been used in previous studies [16,21–29]. The following predictor data were collected at admission: patient age (65–69, 70–74, 75–79, 80–84, 85–89, 90–94, and ≥95 years), sex, BMI (<17.0, 17.0–18.4, 18.5–24.9, 25.0–29.90, and ≥30.0) [30], need for assistance with at least one of the ten activities of daily living (ADL) in the Barthel index (feeding, bathing, grooming, dressing, bowel control, bladder control, toilet use, transfers from bed to chair, mobility on level surfaces, and stairs) [31], malnutrition, consciousness level (using the Japan coma scale: 0: alert; 1–3: delirious; 10–30: somnolent; and 100–300: coma), emergency hospitalization, previous unplanned hospitalization for aspiration pneumonia within the last 90 days at the same hospital, and comorbidities. In the DPC data, malnutrition is defined as a serum albumin level of 3.0 g/dL or less, parenteral nutrition, or tube feeding at admission. Comorbidities included congestive heart failure, diabetes mellitus, metastatic cancer, cerebrovascular disease, dementia, pressure ulcers, and pleural effusion. In addition, the following procedures were used as predictors and had been collected no later than the day after admission: oxygen administration, respiratory support (high-flow nasal cannula, noninvasive ventilation, and invasive mechanical ventilation), vasopressor administration, and sputum suctioning.

### Development of the models

Multilevel logistic regression models were developed in the derivation cohort using the hospital identifiers as random intercepts [32]. Home discharge was the outcome variable for the cases admitted from home; home/nursing home discharge was the outcome variable for cases admitted from a nursing home. The 19 predictors described above constituted the explanatory variables.

### Validation of the models

The predictive performance of our models in terms of discrimination was assessed using the area under curve (AUC) of the receiver operating characteristic (ROC) curve, while calibration was assessed using the intercept and slope obtained from a calibration plot. Internal validity was assessed with a bootstrapping bias correction approach to adjust the optimism in our prediction models [20,33]. We created 200 bootstrap samples by drawing with replacements from the derivation cohort. We calculated the performance measures for each bootstrap sample, including the AUC and calibration slope. To obtain the optimism-corrected performance estimate, we calculated the differences between the performance measures in each bootstrap sample and their performance measures in the derivation cohort.

For internal-external validation, our prediction models were applied to the validation cohort. The predictive performance in terms of discrimination was examined using the AUC, while calibration was examined using the intercept and slope obtained from the calibration plot.

### Development of probability scores and nomograms

To facilitate prediction of the individual probabilities of home/nursing home discharge, we developed probability scores. The regression coefficients were divided by 0.3 and rounded to the nearest integer to assign weights [34,35]. The predictor weights were added to calculate the total probability score. To validate the probability scores, we calibrated the scores by plotting the mean predicted and observed probabilities of the outcomes for cases with the same scores in the validation cohort. Scores were merged into adjacent scores where the number of cases accounted for less than 0.5% of the population [34]. We also created nomograms to facilitate the use of the probability scores in clinical practice.

All the statistical analyses were performed using R software version 4.0.2 (R Foundation for Statistical Computing, Vienna, Austria). A two-sided significance level of 0.05 was used. The study was approved by the Ethics Committee of the Kyoto University Graduate School of Medicine (reference number R0135) and was conducted in accordance with the Ethical Guidelines for Medical and Health Research Involving Human Subjects of the Ministry of Health, Labour and Welfare, Japan. Because of the anonymous nature of the data, the requirement for informed consent was waived.

## Results

**Fig 1:**
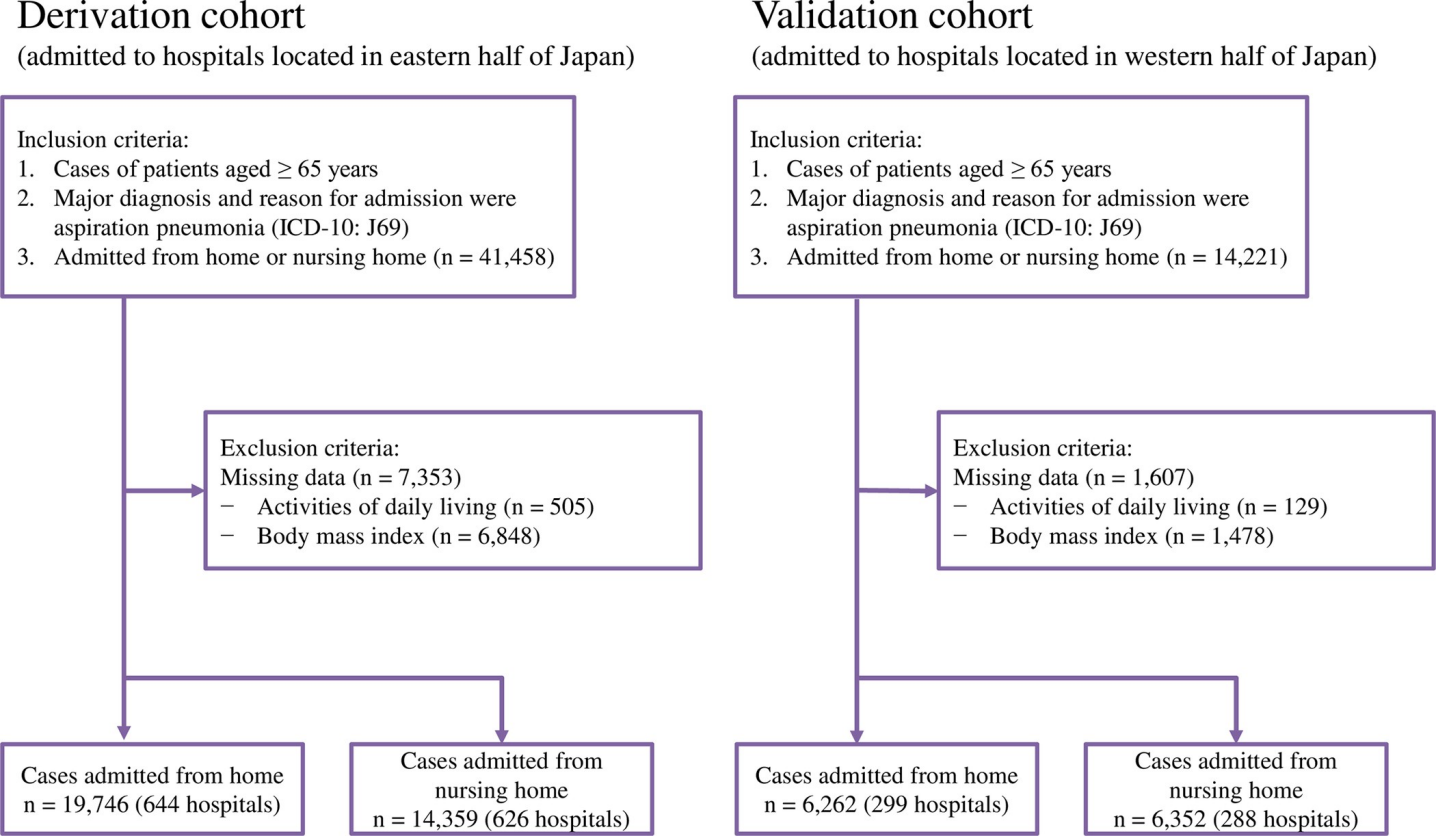
Flow chart of case selection.

In the derivation cohort, we identified 19,746 cases admitted from home to one of 644 hospitals, and 14,359 cases admitted from a nursing home to one of 626 hospitals. In the validation cohort, we identified 6,262 cases admitted from home to one of 299 hospitals, and 6,352 cases admitted from a nursing home to one of 288 hospitals. Fig 1 shows the case selection process.

Table 1 presents the baseline characteristics and outcomes of the derivation and validation cohorts. Although almost all the characteristics and outcomes of both cohorts were similar, the validation cohort had lower rates of emergency hospitalization and in-hospital mortality than the derivation cohort. Compared to the groups admitted from home, the groups admitted from a nursing home were older and included fewer males. They also had lower BMI, more assistance needs (with at least one of the ten ADL functions of the Barthel index), more malnutrition, more frequent emergency hospitalization, more congestive heart failure, more cerebrovascular disease, more dementia, more pressure ulcers, more pleural effusion, more oxygen administration, more sputum suctioning, longer lengths of stay, and more in-hospital mortality.

**Table 1:** Baseline characteristics and outcomes of the derivation and validation cohorts.

Characteristics	Derivation cohort		Validation cohort	
	Cases admitted from home	Cases admitted from nursing home	Cases admitted from home	Cases admitted from nursing home
	(n = 19,746)	(n = 14,359)	(n = 6,262)	(n = 6,352)
Age, mean (SD)	84.63 (7.56)	86.63 (7.43)	84.74 (7.87)	87.16 (7.37)
Male, n (%)	12884 (65.2)	7111 (49.5)	4198 (67.0)	3108 (48.9)
BMI, median [IQR]	18.94 [16.55, 21.63]	18.06 [15.94, 20.41]	19.12 [16.66, 21.83]	18.02 [15.86, 20.45]
Assistance needs with ≥ 1 ADL functions, n (%)	18271 (92.5)	14187 (98.8)	5767 (92.1)	6309 (99.3)
Malnutrition, n (%)	7074 (35.8)	6745 (47.0)	2415 (38.6)	3259 (51.3)
Consciousness level, n (%)				
Japan coma scale: 0	10598 (53.7)	5469 (38.1)	3370 (53.8)	2134 (33.6)
Japan coma scale: 1–3	6635 (33.6)	5720 (39.8)	2133 (34.1)	2582 (40.6)
Japan coma scale: 10–30	1728 (8.8)	2308 (16.1)	538 (8.6)	1168 (18.4)
Japan coma scale: 100–300	785 (4.0)	862 (6.0)	221 (3.5)	468 (7.4)
Emergency hospitalization, n (%)	14518 (73.5)	10654 (74.2)	3343 (53.4)	3738 (58.8)
Hospitalization for aspiration pneumonia within 90 days, n (%)	1584 (8.0)	1319 (9.2)	675 (10.6)	675 (10.6)
Comorbidities, n (%)				
Congestive heart failure	3110 (15.8)	2317 (16.1)	1076 (17.2)	1179 (18.6)
Diabetes mellitus	3472 (17.6)	1985 (13.8)	1126 (18.0)	883 (13.9)
Metastatic cancer	327 (1.7)	90 (0.6)	91 (1.5)	35 (0.6)
Cerebrovascular disease	3893 (19.7)	3486 (24.3)	1538 (24.6)	2002 (31.5)
Dementia	5070 (25.7)	5755 (40.1)	1579 (25.2)	2658 (41.8)
Pressure ulcers	543 (2.7)	569 (4.0)	153 (2.4)	210 (3.3)
Pleural effusion	1108 (5.6)	863 (6.0)	296 (4.7)	355 (5.6)
Oxygen administration, n (%)	11958 (60.6)	9336 (65.0)	3602 (57.5)	4191 (66.0)
Respiratory support, n (%)	486 (2.5)	253 (1.8)	227 (3.6)	179 (2.8)
Vasopressor administration, n (%)	500 (2.5)	353 (2.5)	191 (3.1)	211 (3.3)
Sputum suctioning, n (%)	8194 (41.5)	8038 (56.0)	2217 (35.4)	3472 (54.7)
Outcomes				
Home discharge, n (%)	10760 (54.5)	–	3480 (55.6)	–
Discharge to nursing home or home, n (%)	–	7071 (49.2)	–	3250 (51.2)
Length of stay, median [IQR]	18 [11, 31]	20 [13, 33]	17 [10, 29]	18 [12, 30]
In-hospital mortality, n (%)	3324 (16.8)	2879 (20.1)	774 (12.4)	1118 (17.6)

ADL were defined as the 10 components of the Barthel index (feeding, bathing, grooming, dressing, bowel control, bladder control, toilet use, transfers from bed to chair, mobility on level surfaces, and stairs). Malnutrition was defined as a serum albumin level of 3.0 g/dL or less, parenteral nutrition, or tube feeding at admission. Respiratory support included high-flow nasal cannula, noninvasive ventilation, and invasive mechanical ventilation. Abbreviations: SD, standard deviation; IQR, interquartile range; BMI, body mass index; ADL, activities of daily living.

### Prediction models according to residence before admission

Table 2 presents the results of the multilevel logistic regression analyses of the outcomes for the derivation cohort. In the prediction model for cases admitted from home, older age, low BMI, needing assistance with at least one of the ten ADL functions of the Barthel index, impaired level of consciousness on the Japan coma scale, metastatic cancer, respiratory support, vasopressor administration, and sputum suctioning were strong predictors of home discharge. In the prediction model for cases admitted from a nursing home, male, impaired level of consciousness on the Japan coma scale, metastatic cancer, respiratory support, and vasopressor administration were strong predictors of home/nursing home discharge.

**Table 2:** Regression coefficients, odds ratios, and derived weights for each predictor of the two outcomes according to residence before admission in the derivation cohort.

	Home discharge of cases admitted from home			Home/nursing home discharge of cases admitted from nursing home		
	Coef- ficient	Odds ratio (95% CI)	Weight	Coef- ficient	Odds ratio (95% CI)	Weight
Intercept	2.71			1.09		
Age (reference: 65–69 years)						
70–74 years	−0.01	0.99 (0.80–1.23)	0	0.23	1.25 (0.95–1.65)	1
75–79 years	−0.32	0.73 (0.60–0.88)	−1	−0.23	0.80 (0.61–1.03)	−1
80–84 years	−0.43	0.65 (0.54–0.79)	−1	−0.23	0.80 (0.62–1.02)	−1
85–89 years	−0.60	0.55 (0.46–0.67)	−2	−0.24	0.79 (0.62–1.00)	−1
90–94 years	−0.61	0.54 (0.45–0.66)	−2	−0.23	0.79 (0.62–1.01)	−1
≥ 95 years	−0.77	0.46 (0.38–0.57)	−3	−0.23	0.79 (0.62–1.03)	−1
Male	−0.23	0.79 (0.74–0.85)	−1	−0.51	0.60 (0.56–0.65)	−2
BMI (reference: 18.5 ≤ BMI < 25.0)						
< 17.0	−0.55	0.58 (0.54–0.62)	−2	−0.33	0.72 (0.66–0.78)	−1
17.0 ≤ BMI < 18.5	−0.27	0.77 (0.70–0.84)	−1	−0.10	0.90 (0.82–1.00)	0
25.0 ≤ BMI < 30	0.21	1.24 (1.08–1.41)	1	0.05	1.05 (0.86–1.27)	0
≥ 30	0.26	1.30 (0.91–1.86)	1	0.05	1.05 (0.57–1.92)	0
Assistance needs with ≥ 1 ADL functions	−0.82	0.44 (0.38–0.51)	−3	0.18	1.19 (0.85–1.66)	1
Malnutrition	−0.38	0.68 (0.64–0.73)	−1	−0.32	0.73 (0.67–0.78)	−1
Japan coma scale (reference: 0)						
1–3	−0.31	0.73 (0.68–0.79)	−1	−0.11	0.90 (0.82–0.98)	0
10–30	−0.54	0.58 (0.52–0.66)	−2	−0.28	0.75 (0.67–0.84)	−1
100–300	−1.17	0.31 (0.26–0.37)	−4	−0.55	0.58 (0.49–0.68)	−2
Emergency hospitalization	−0.13	0.87 (0.81–0.95)	0	−0.03	0.97 (0.88–1.06)	0
Hospitalization for aspiration pneumonia within 90 days	0.45	1.56 (1.39–1.76)	1	0.03	1.03 (0.91–1.17)	0
Comorbidities						
Congestive heart failure	0.02	1.02 (0.93–1.11)	0	−0.09	0.91 (0.82–1.00)	0
Diabetes mellitus	0.11	1.12 (1.03–1.22)	0	0.07	1.07 (0.97–1.19)	0
Metastatic cancer	−0.56	0.57 (0.45–0.73)	−2	−1.18	0.31 (0.18–0.52)	−4
Cerebrovascular disease	0.09	1.09 (1.01–1.18)	0	0.01	1.01 (0.93–1.10)	0
Dementia	−0.15	0.86 (0.80–0.93)	−1	0.16	1.17 (1.08–1.26)	1
Pressure ulcers	−0.31	0.73 (0.60–0.89)	−1	−0.42	0.66 (0.55–0.79)	−1
Pleural effusion	−0.37	0.69 (0.60–0.79)	−1	−0.24	0.79 (0.67–0.92)	−1
Oxygen administration	−0.38	0.68 (0.64–0.73)	−1	−0.33	0.72 (0.66–0.78)	−1
Respiratory support	−0.83	0.43 (0.35–0.55)	−3	−1.11	0.33 (0.24–0.46)	−4
Vasopressor administration	−0.79	0.45 (0.36–0.57)	−3	−0.64	0.53 (0.41–0.68)	−2
Sputum suctioning	−0.53	0.59 (0.55–0.63)	−2	−0.41	0.67 (0.62–0.72)	−1

ADL were defined as the 10 components of the Barthel index (feeding, bathing, grooming, dressing, bowel control, bladder control, toilet use, transfers from bed to chair, mobility on level surfaces, and stairs). Malnutrition was defined as a serum albumin level of 3.0 g/dL or less, parenteral nutrition, or tube feeding at admission. Respiratory support included high-flow nasal cannula, noninvasive ventilation, and invasive mechanical ventilation. Abbreviations: CI, confidence interval; BMI, body mass index; ADL, activities of daily living.

### Validation of the models

**Fig 2:**
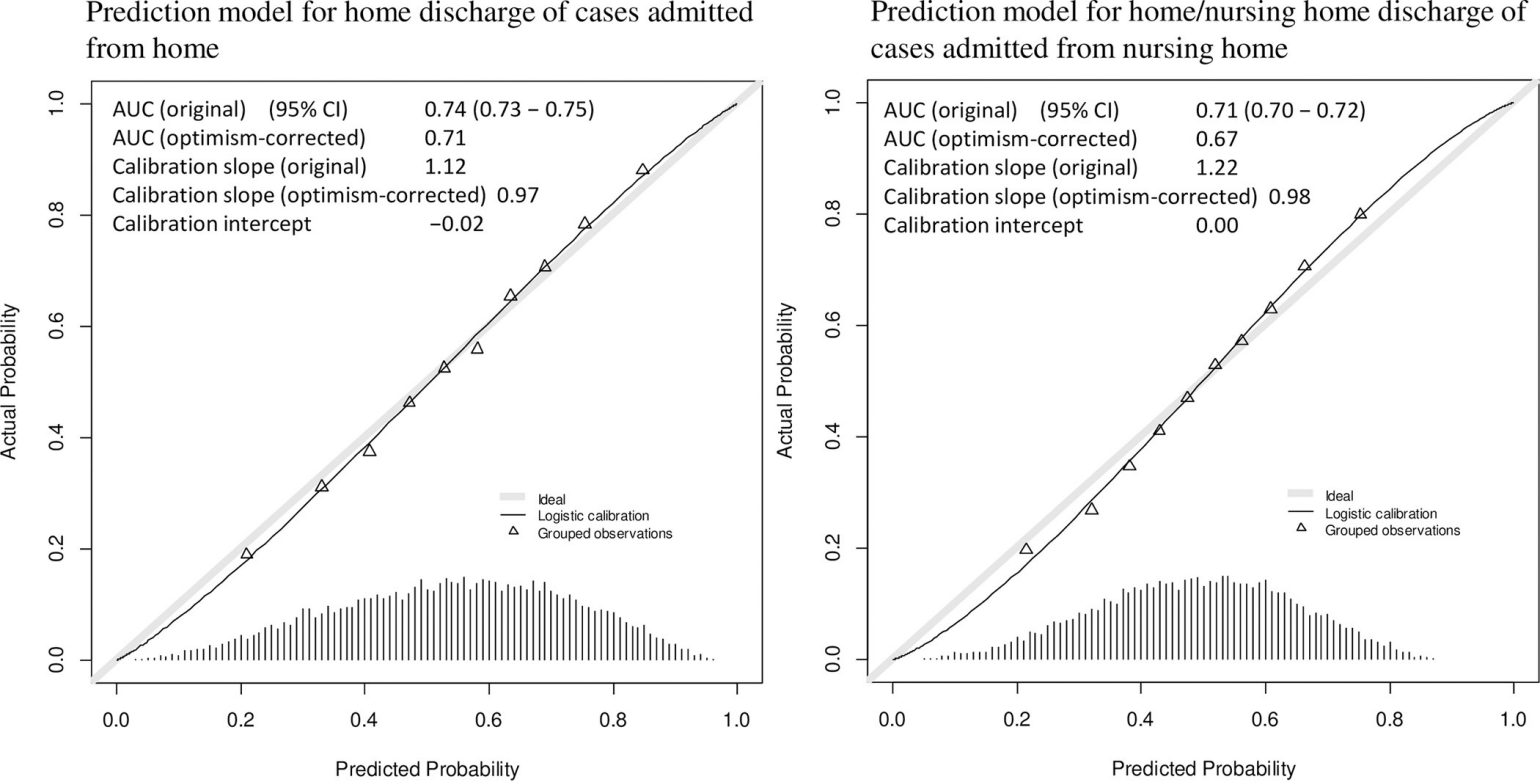
Results of internal validation with calibration plots of the two models. AUC, area under curve; 95% CI, confidence interval.

Fig 2 presents the internal validation results for the two models. In the model for the home discharge of cases admitted from home, the original AUC, calibration slope, and calibration intercept were 0.74 (95% confidence interval [CI], 0.73–0.75), 1.12, and −0.02, respectively. In the internal validation, the optimism-corrected AUC and calibration slope were 0.71 and 0.97, respectively. In the model for the home/nursing home discharge of cases admitted from a nursing home, the original AUC, calibration slope, and calibration intercept were 0.71 (95% CI, 0.70–0.72), 1.22, and 0.00, respectively. In the internal validation, the optimism-corrected AUC and calibration slope were 0.67 and 0.98, respectively. The discrimination and calibration of the model for cases admitted from a nursing home were lower than those of the model for cases admitted from home.

**Fig 3:**
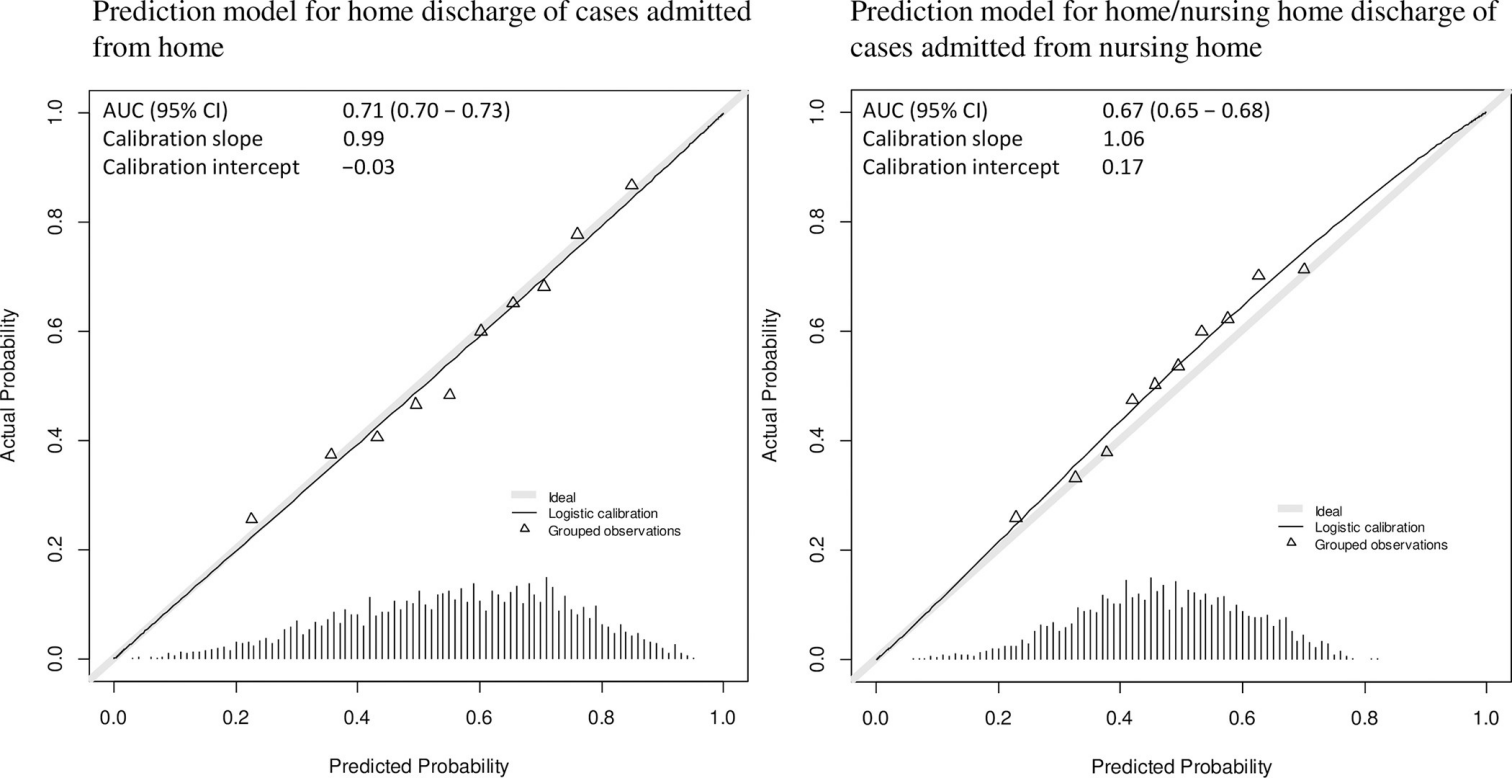
Results of internal-external validation with calibration plots of the two models. AUC, area under curve; 95% CI, confidence interval.

Fig 3 presents the internal-external validation results for the two models. In the internal-external validation of the model for cases admitted from home, the AUC, calibration slope, and calibration intercept were 0.71 (95% CI, 0.70–0.73), 0.99, and −0.03, respectively. Regarding the model for cases admitted from a nursing home, the AUC, calibration slope, and calibration intercept were 0.67 (95% CI, 0.65–0.68), 1.06, and 0.17, respectively. The discrimination and calibration of the model for cases admitted from a nursing home were slightly worse than those of the model for cases admitted from home.

### Probability scores

**Fig 4:**
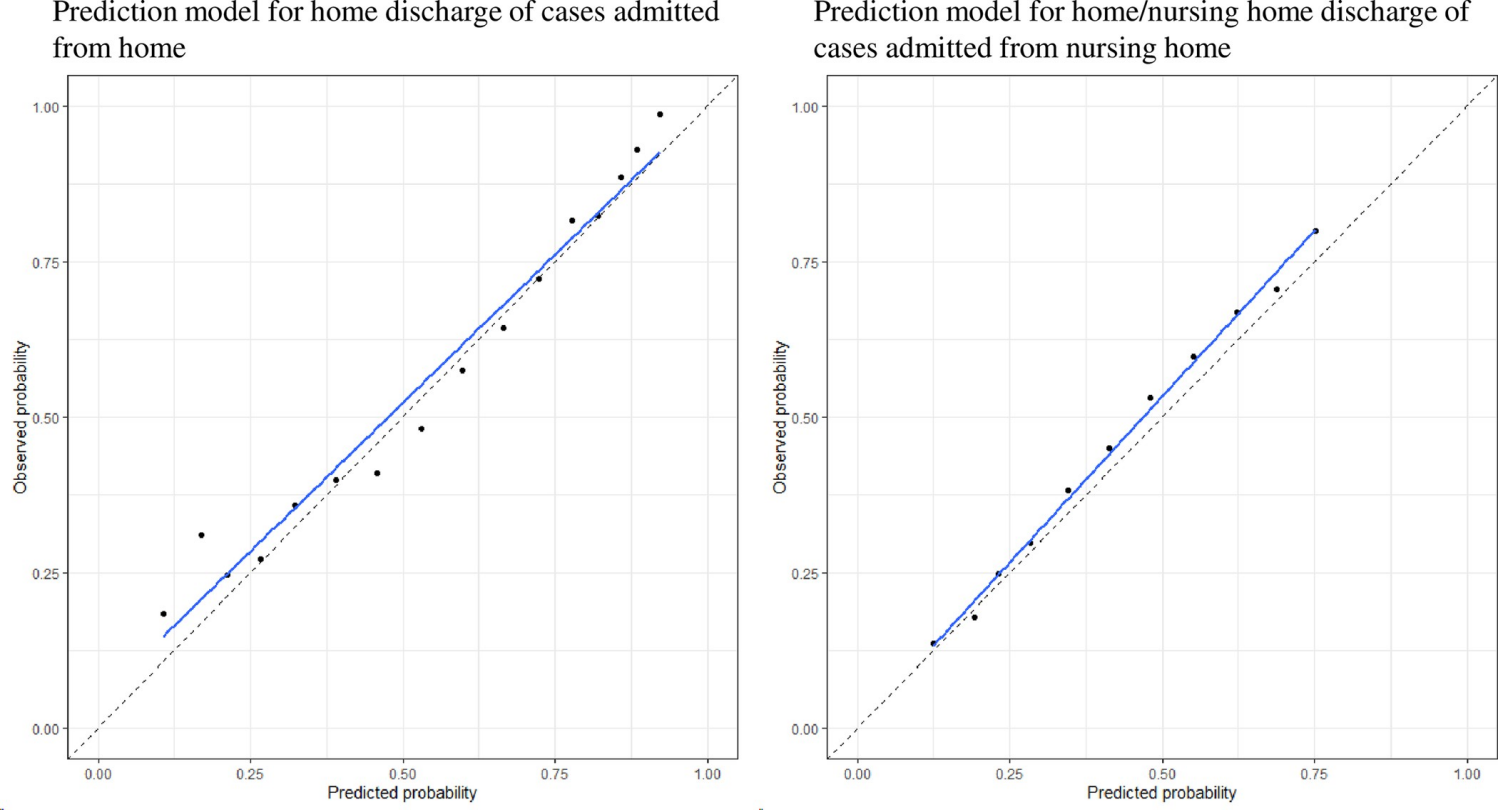
Results of validation using calibration plots of probability scores of the two models. The dots represent each probability score. The blue lines are the regression lines through the dots of the probability scores.

The probability scores are shown in Table 2. The validation results using the calibration plots of the probability scores of the two models in the validation cohort are shown in Fig 4. The probability scores of the two models were able to predict each outcome.

**Fig 5:**
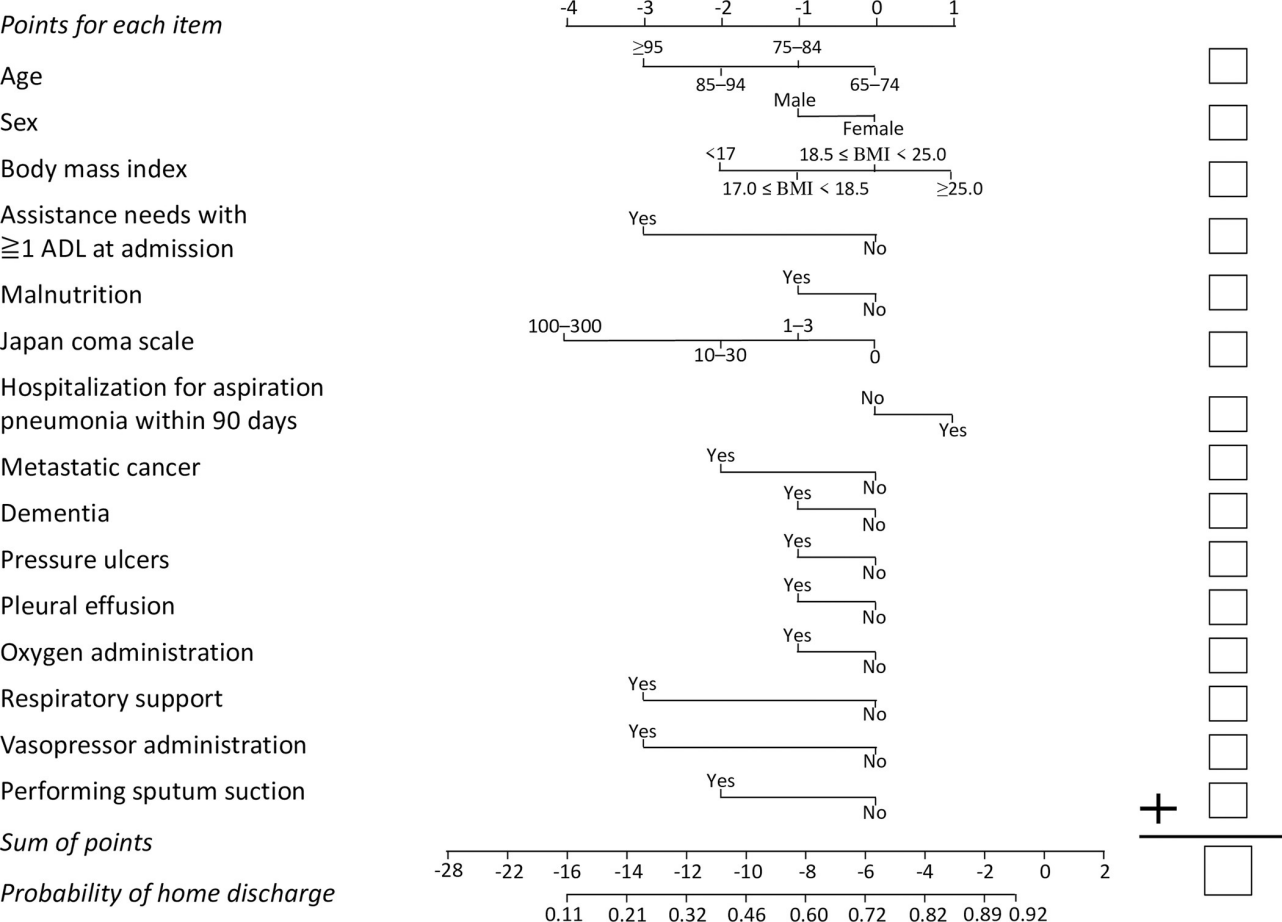
Nomogram for estimating the probability of home discharge for cases admitted from home. Draw a vertical line upward from the corresponding value of each predictor to the points bar to obtain the points for each item. Based on the sum of the points for each item, draw a vertical line downward from the corresponding sum of the points to calculate the probability of home discharge. ADL were defined as the 10 components of the Barthel index (feeding, bathing, grooming, dressing, bowel control, bladder control, toilet use, transfers from bed to chair, mobility on level surfaces, and stairs). Malnutrition was defined as a serum albumin level of 3.0 g/dL or less, parenteral nutrition, or tube feeding at admission. Respiratory support included high-flow nasal cannula, noninvasive ventilation, and invasive mechanical ventilation.

**Fig 6:**
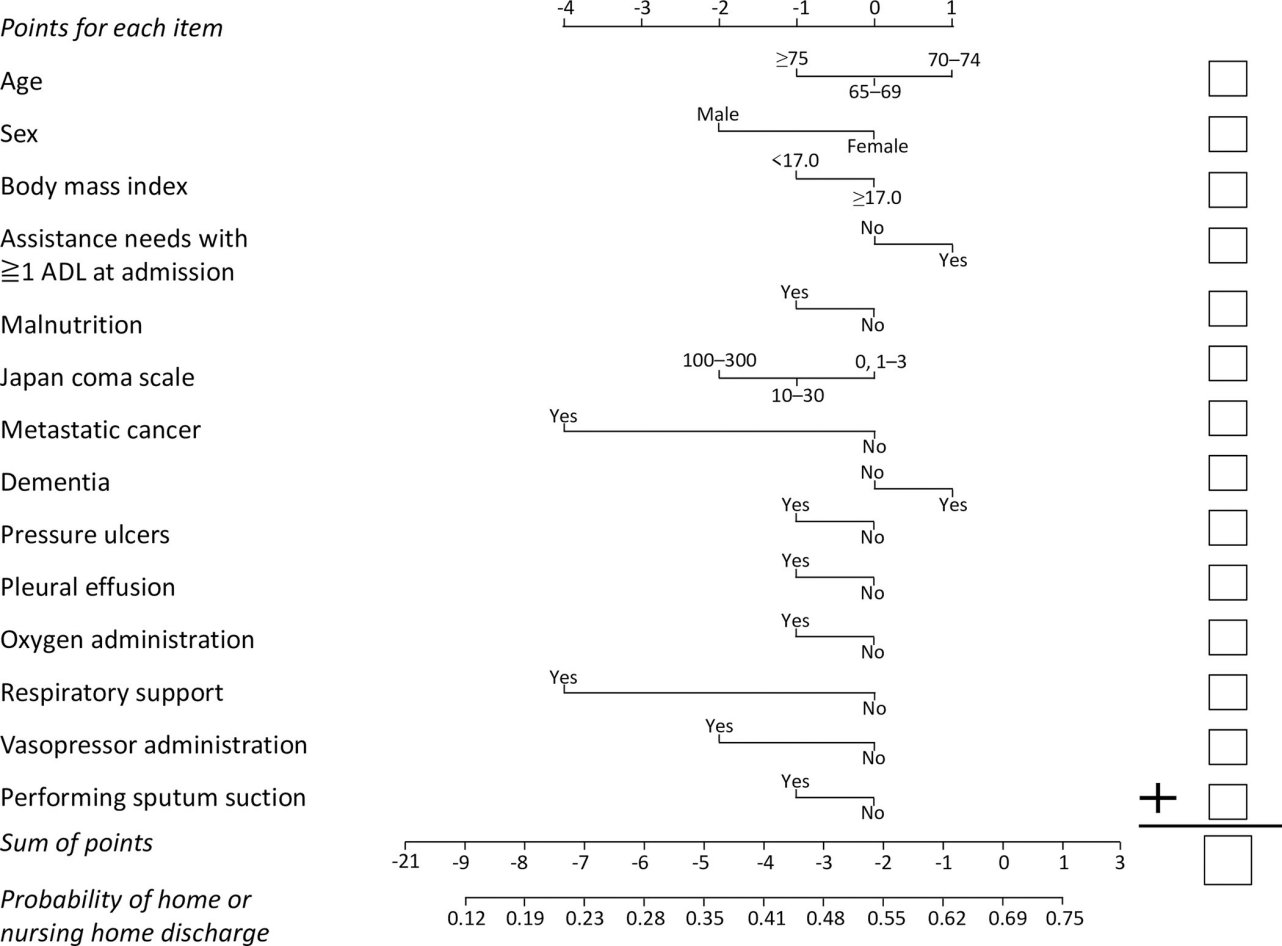
Nomogram for estimating the probability of home/nursing home discharge for cases admitted from a nursing home. Draw a vertical line upward from the corresponding value of each predictor to the points bar to obtain the points for each item. Based on the sum of the points for each item, draw a vertical line downward from the corresponding sum of the points to calculate the probability of home/nursing home discharge. ADL were defined as the 10 components of the Barthel index (feeding, bathing, grooming, dressing, bowel control, bladder control, toilet use, transfers from bed to chair, mobility on level surfaces, and stairs). Malnutrition was defined as a serum albumin level of 3.0 g/dL or less, parenteral nutrition, or tube feeding at admission. Respiratory support included high-flow nasal cannula, noninvasive ventilation, and invasive mechanical ventilation.

Fig 5 presents the nomogram for estimating the probability of home discharge for cases admitted from home; Fig 6 presents the nomogram for estimating the probability of home/nursing home discharge for cases admitted from a nursing home.

## Discussion

Using a nationwide inpatient database, we developed and validated two models to predict the discharge destination of elderly patients with aspiration pneumonia using baseline characteristics that could be obtained within one day after a patient’s admission. One model was for patients admitted to hospital from home; the other was for patients admitted to hospital from a nursing home. The prediction model for the home discharge of cases admitted from home had an AUC of 0.71, a calibration slope of 0.99, and a calibration intercept of −0.03 in its internal-external validation. The prediction model for the home/nursing home discharge of cases admitted from a nursing home had an AUC of 0.67, a calibration slope of 1.06, and a calibration intercept of 0.17 in the internal-external validation. The nomograms created here for estimating the probability of home/nursing home discharge could be easily used in clinical practice.

The proposed prediction models and nomograms can be used to facilitate the implementation of early discharge planning by identifying patients who are unlikely to be discharged to either their home or a nursing home. That the fact our models are based on nationwide multi-center data is a particular strength of the study. In addition, all the predictor values can be collected within one day of a patient’s admission. Thus, for example, if a patient is admitted from home and has less than a 50% probability of home discharge (based on the nomogram for those admitted from home, which consists of baseline patient characteristics determined within one day after admission), discharge planning could begin as early as the day after admission. Furthermore, patients hospitalized for aspiration pneumonia and not discharged to their home or to a nursing home, i.e., those discharged to a special type of nursing home that provides medical care or to a hospital with long-term care beds, have lower functional and nutritional status at discharge [7,36]. Poor functional and nutritional status is associated with six-month and one-year mortality in patients with aspiration pneumonia [21,22]. For patients who are unlikely to be discharged to their home or to a nursing home based on their nomograms, healthcare staff may need to initiate a discussion of end-of-life care with the patient and the patient’s family.

The AUC of the prediction model for the home/nursing home discharge of cases admitted from a nursing home was lower than the AUC of the prediction model for the home discharge of cases admitted from home. In the calibration plots, the range of probabilities of home/nursing home discharge based on the prediction model for cases admitted from a nursing home was narrower than the range of probabilities of home discharge based on the prediction model for cases admitted from home. The lower AUC and calibration of the predictive model for cases admitted from a nursing home may have been due to a similarity of the combination of baseline characteristics among cases admitted from nursing homes. For example, many cases admitted from nursing homes may have been older than 85 and female, had a BMI of less than 17.0, and needed assistance with at least one of the ten ADL functions of the Barthel index.

Our study had several limitations. First, because there is no gold standard test for detecting aspiration, patients whose diagnosis of aspiration pneumonia was uncertain might have been included. However, validation studies identifying aspiration pneumonia using an administrative database have been conducted [5,37,38], and the use of ICD-10 codes to identify patients with aspiration pneumonia has been validated [39]. Second, information on patients’ social factors could not be included in the analysis because of the nature of the DPC data. Discharge destinations may be affected by social factors such as family structure [40,41] and home environment [42], especially in cases admitted from home. Thus, including these variables in the model could improve their predictive performance.

## Conclusions

Using a nationwide inpatient database, we developed and validated two novel models to predict home/nursing home discharge of elderly patients with aspiration pneumonia based on baseline characteristics that can be obtained within one day after admission to hospital. We also created nomograms that could be used easily in clinical practice. Use of these models and nomograms can facilitate the early implementation of discharge planning.
